# Supplementary material for: Causal Relationship between Aging and Anorexia Nervosa: A White-Matter-Microstructure-Mediated Mendelian Randomization Analysis
Source: Biomedicines. 2024 Aug 16;12(8):1874. doi: 10.3390/biomedicines12081874 (PMC11351342; doi:10.3390/biomedicines12081874)
Supplement: Supplementary file 1 [file biomedicines-12-01874-s001.zip › Supplementary Figure/Figure S4/Singe SNP plot of MR analysis for TL to ubm-b-1858.pdf]

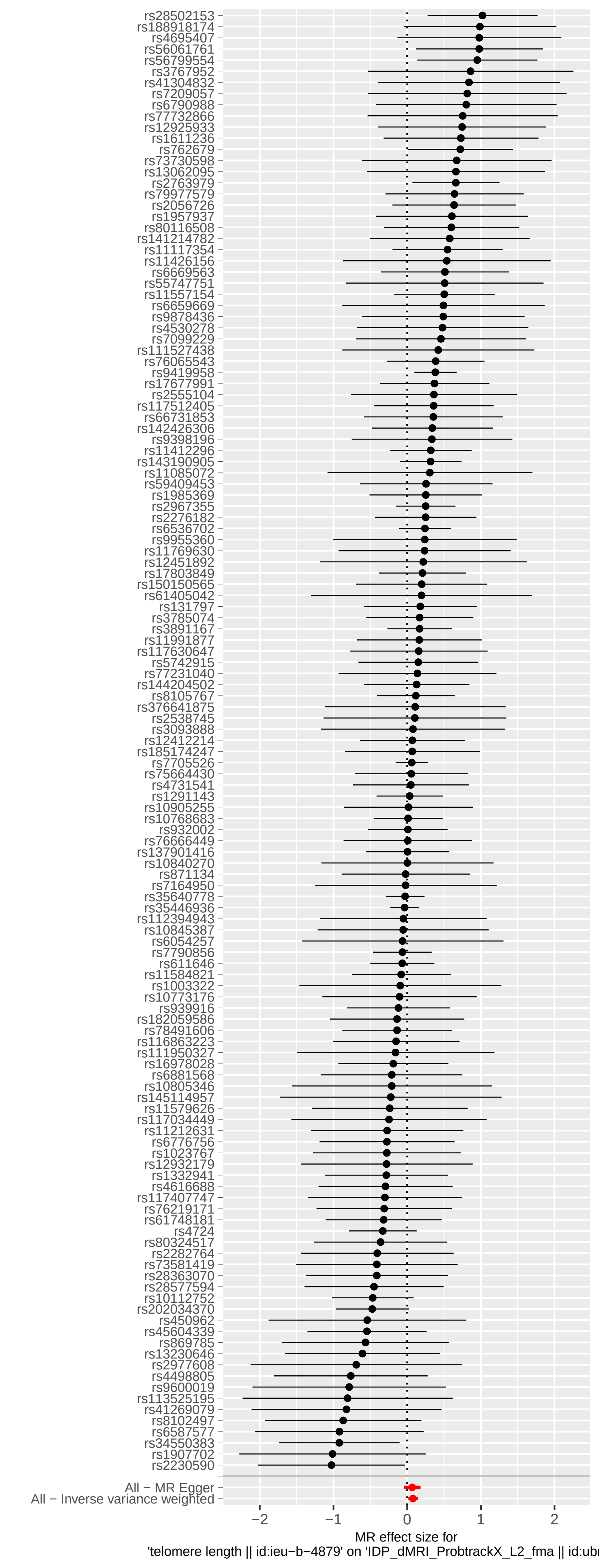
rs28502153
rs188918174
rs4695407
rs56061761
rs56799554
rs3767952
rs41304832
rs7209057
rs6790988
rs77732866
rs12925933
rs1611236
rs762679
rs73730598
rs13062095
rs2763979
rs79977579
rs2056726
rs1957937
rs80116508
rs141214782
rs11117354
rs11426156
rs6669563
rs55747751
rs11557154
rs6659669
rs9878436
rs4530278
rs7099229
rs111527438
rs76065543
rs9419958
rs17677991
rs2555104
rs117512405
rs66731853
rs142426306
rs9398196
rs11412296
rs143190905
rs11085072
rs59409453
rs1985369
rs2967355
rs2276182
rs6536702
rs9955360
rs11769630
rs12451892
rs17803849
rs150150565
rs61405042
rs131797
rs3785074
rs3891167
rs11991877
rs117630647
rs5742915
rs77231040
rs144204502
rs8105767
rs376641875
rs2538745
rs3093888
rs12412214
rs185174247
rs7705526
rs75664430
rs4731541
rs1291143
rs10905255
rs10768683
rs932002
rs76666449
rs137901416
rs10840270
rs871134
rs7164950
rs35640778
rs35446936
rs112394943
rs10845387
rs6054257
rs7790856
rs611646
rs11584821
rs1003322
rs10773176
rs939916
rs182059586
rs78491606
rs116863223
rs111950327
rs16978028
rs6881568
rs10805346
rs145114957
rs11579626
rs117034449
rs11212631
rs6776756
rs1023767
rs12932179
rs1332941
rs4616688
rs117407747
rs76219171
rs61748181
rs4724
rs80324517
rs2282764
rs73581419
rs28363070
rs28577594
rs10112752
rs202034370
rs450962
rs45604339
rs869785
rs13230646
rs2977608
rs4498805
rs9600019
rs113525195
rs41269079
rs8102497
rs6587577
rs34550383
rs1907702
rs2230590
All − MR Egger
All − Inverse variance weighted
−2
−1
0
1
2
MR effect size for
'telomere length || id:ieu−b−4879' on 'IDP_dMRI_ProbtrackX_L2_fma || id:ub
